# A new species of the *Marphysa sanguinea* complex from French waters (Bay of Biscay, NE Atlantic) (Annelida, Eunicidae)

**DOI:** 10.3897/zookeys.716.14070

**Published:** 2017-11-23

**Authors:** Nicolas Lavesque, Guillemine Daffe, Paulo Bonifácio, Pat Hutchings

**Affiliations:** 1 Univ. Bordeaux, EPOC, UMR 5805, Station Marine d’Arcachon, 2 Rue du Professeur Jolyet, 33120 Arcachon, France; 2 CNRS, EPOC, UMR 5805, Station Marine d’Arcachon, 2 Rue du Professeur Jolyet, 33120 Arcachon, France; 3 Ifremer, Centre Bretagne, REM EEP, Laboratoire Environnement Profond, ZI de la Pointe du Diable, CS 10070, F-29280 Plouzané, France; 4 Australian Museum Research Institute,; 5 Australian Museum, 1, William Street, Sydney, NSW 2010, Australia

**Keywords:** Bait worms, eastern Atlantic, France, Marphysa, molecular, morphology, Polychaeta, taxonomy

## Abstract

A new species of Eunicidae, *Marphysa victori* **sp. n.**, has been identified from Arcachon Bay, Bay of Biscay, NE Atlantic. This new species, belonging to the *sanguinea* complex, is characterised by branchiae with long filaments from chaetigers 26–34, the presence of four types of pectinate chaetae with first ones present from chaetiger 2, a large number of both pectinate chaetae and compound spinigers, and the pygidium with only one pair of pygidial cirri. An identification key for European species of the genus *Marphysa* is provided.

## Introduction

In Arcachon Bay, blood worms of the genus *Marphysa* Quatrefages, 1866 are widely collected as bait both by recreational and professional fishermen. Since 2011, 13 companies (with a total of 26 employees) were operating in the lagoon and they recorded that 1.3–2.5 tons/year (wet weight) of *Marphysa* were collected which represents approximatively 400,000 worms. In reality, around 1 million of these worms could be fished each year in the bay. Most of these worms are shipped alive by air (in boxes with plant litter) to sellers situated on the western French Mediterranean coasts. Then, they are sold to recreational fishermen and used locally. Until now, these blood worms were misidentified as *Marphysa sanguinea* (Montagu, 1813), which was originally described from the south coast of Devon, UK (Hutchings and Karageorgopoulos 2003).

The family Eunicidae Berthold, 1827 is a very speciose family with nine genera and with more than 400 valid species distributed worldwide (Zanol and Read 2012). The genus *Marphysa* comprises around 81 nominal species with five valid species only known from European waters (Read and Bellan 2016): *Marphysa bellii* (Audouin & Milne Edwards, 1833), *Marphysa fallax* Marion & Bobretzky, 1875, *Marphysa kinbergi* McIntosh, 1910, *Marphysa sanguinea* and *Marphysa totospinata* Lu & Fauchald, 1998. *Marphysa saxicola* Langerhans, 1881 was recently transferred to the genus *Nicidion* Kinberg, 1865 (Arias and Nuñez 2016) and *Marphysa simplex* (Langerhans, 1884) should be regarded as an invalid species (Gil 2011). Finally, we consider that *Marphysa grunwaldi* (Risso, 1826) and *Marphysa triantennata* (Risso, 1826), described from French Mediterranean Sea, should be considered as *nomen nuda* because of very brief descriptions, lack of any figures, and absence of type material.

According to Fauchald (1970), species of the genus *Marphysa* can be grouped into four artificial groups based on the type of compound chaetae: no compound chaetae present (Group A), only compound spinigers present (Group B), only compound falcigers present (Group C) and both compound spinigers and falcigers present (Group D). Glasby and Hutchings (2010) added a fifth group, having compound spinigers only anteriorly and posterior segments only with simple limbate chaetae, and also encapsulating embryos in jelly cocoons. Each group can also then be divided into species having branchiae present only on anterior parapodia (subdivision 1) or branchiae present over most of the body (subdivision 2). In European waters, *M. sanguinea* is the only representative of the Group B2 currently reported.

*Marphysa sanguinea* has been recorded in Arcachon Bay by numerous workers (Lafont 1871; Boisseau 1962; Auby 1991; Blanchet 2004; Salvo 2010). However, all these papers just list the fauna present as a result of ecological studies without commenting on the species. It appears that no material was deposited in a museum and so could not be examined for this study. These records are not surprising as this species is originally described from the northern coast of the English Channel. Fauvel (1923) in his monograph of French polychaetes lists *M. sanguinea* and provides description and illustrations but without commenting on what material he examined. Nevertheless, recent re-descriptions of this species (Hutchings and Karageorgopoulos 2003; Hutchings et al. 2012) suggest that records of this species from outside the type locality should be checked, and that many records have been misidentified and represent new species (Zanol et al. 2016, 2017). Hutchings and Kupriyanova (2017) encourage taxonomists to check carefully their specimens.

Both morphological and molecular analyses confirm the existence of an undescribed species of *Marphysa* in Arcachon Bay. The present paper provides the description of this species as well as a key for European described species of this genus.

## Materials and methods

### Sampling and morphological analyses

**Figure 1.**
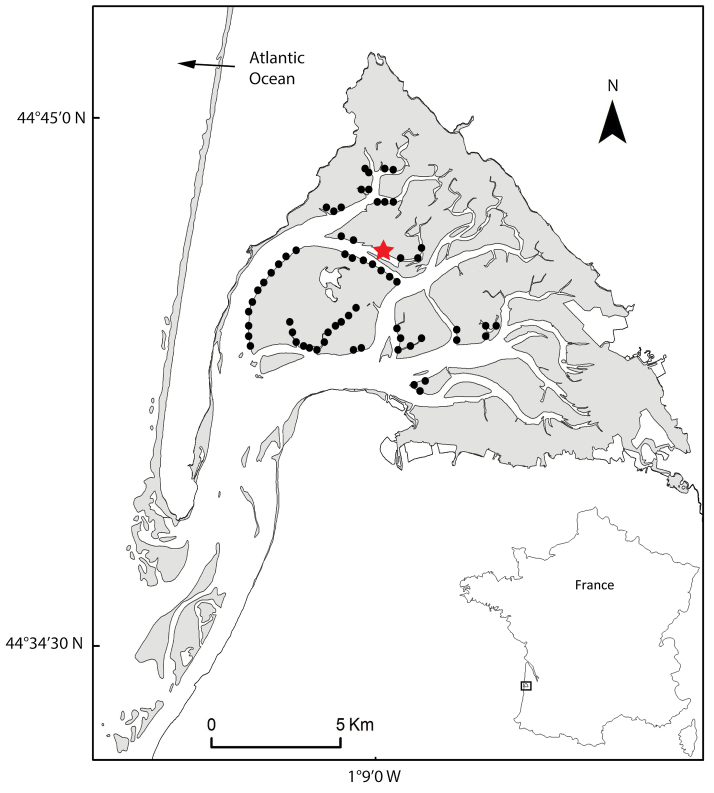
Sampling sites of *Marphysa victori* sp. n. in Arcachon Bay (Bay of Biscay, western France). Black dots: presence (according to fishermen); red star: type locality.

Specimens examined in this study were collected in Arcachon Bay (Fig. 1) in September 2016 by hand, using a shovel and spade from intertidal mud flats. Live specimens were anaesthetized with 7% magnesium chloride (MgCl_2_). A small piece of body was removed from several specimens and fixed in 96% ethanol for molecular studies. The rest of each specimen was fixed in 4% formaldehyde seawater solution, then transferred to 70% ethanol for morphological analyses. Preserved specimens were examined under a Nikon SMZ25 stereomicroscope and a Nikon Eclipse E400 microscope, and photographed with a Nikon DS-Ri 2 camera. Measurements were made with the NIS-Elements Analysis software. Drawings were made from pictures using Inkscape software and Wacom Intuos 5 tablet.

Selected parapodia along the body were removed from the holotype AM W.49047, dehydrated in ethanol, critical point dried, covered with 20 nm of gold, examined under the scanning electron microscope (JEOL JSM 6480LA) and imaged with a secondary detector at Macquarie University, Sydney, Australia. These parapodia included chaetiger 3 and then chaetigers at 20% intervals along the body (chaetigers 83, 163, 243, 323, 403, and 470). Additionally, the following parapodia were removed from holotype (chaetigers 4, 93, 166, 259, 352, and 445) mounted and examined under the light microscope, with permanent slides made.

The studied material is deposited at the Australian Museum, Sydney (**AM**) and the Muséum National d’Histoire Naturelle, Paris (**MNHN**). Additional material is lodged in the collection housed at the Arcachon Marine Station.

### Molecular data and analyses

Sub-samples for DNA analysis were removed from live specimens, placed in ethanol 96% and frozen at –20°C. Extraction of DNA was done with QIAamp DNA Micro Kit (QIAGEN) following protocol supplied by the manufacturers. Approximately 400 bp of 16S and 700 bp of COI (cytochrome c oxidase subunit I) genes were amplified using primers diop16SF (TGCAAAGGTAGCATAATCATTTG) and diop16SR (ACTCAGATCACGTAGGA) for 16S were designed, and polyLCO and polyHCO for COI (Carr et al. 2011).

The PCR (Polymerase Chain Reaction) was realised with Gotaq G2 Flexi DNA Polymerase (PROMEGA), with 50 µL mixtures contained: 10µL of 5X Colorless GoTaq® Reaction Buffer (final concentration of 1X), 1.5 µL of MgCl2 solution (final concentration of 1.5mM), 1 µL of PCR nucleotide mix (final concentration of 0.2 mM each dNTP), 0.5 μl of each primer (final concentration of 1µM), 0.2 µl of GoTaq® G2 Flexi DNA Polymerase (5U/µl), 1 μl template DNA and 33.8 µL of nuclease-free water. The temperature profile was as follows for 16S: 94°C/600s - (94°C/60s-59°C/30s-72°C/90s) *40 cycles - 72°C/600s - 4°C, for COI: 94°C/600s - (94°C/40s-44°C/40s-72°C/60s) *5 cycles - (94°C/40s-51°C/40s-72°C/60s) *35 cycles - 72°C/300s - 4°C. Amplified PCR products were analysed by electrophoresis in a 1 % p/v agarose gel stained with ethidium bromide and were sent to GATC Biotech Company to complete double strain sequencing, using same set of primers as used for PCR.

Overlapping sequence (forward and reverse) fragments were merged into consensus sequences and aligned using Clustal Omega. For COI, sequences were translated into amino acid alignment and checked for stop codons to avoid pseudogenes. The minimum length coverage was around 430bp for 16S and 660 bp for COI. All sequences obtained in this study have been deposited in GenBank (Table 1).

**Table 1.** List of terminal taxa used in molecular analysis, GenBank accession numbers, genes analysed, and voucher specimen catalog numbers.

**Species**	**GenBank accession number**	**Gene**	**Voucher specimen catalog number**
Eunice cf. violaceomaculata Ehlers, 1887	GQ497542 ^1^	COI	
*Palola viridis* Gray in Stair, 1847	GQ497556 ^1^	COI	
*Lysidice ninetta* Audouin & Milne Edwards, 1833	GQ497564 ^1^	COI	
*Leodice rubra* (Grube, 1856)	GQ497528 ^1^	COI	
*Marphysa*			
*M. brevitentaculata* Treadwell, 1921	GQ497548 ^1^	COI	
*M. californica* Moore, 1909	GQ497552 ^1^	COI	
*M. disjuncta* Hartman, 1961	GQ497549 ^1^	COI	
*M. regalis* Verrill, 1900	GQ497562 ^1^	COI	
*M. sanguinea* (Montagu, 1813)	GQ497547 ^1^	COI	
*M. sanguinea* (Montagu, 1813)	GQ478157 ^1^	16S	
*M. viridis* Treadwell, 1917	GQ497553 ^1^	COI	
*M. bifurcata* Kott, 1951	KX172177 ^2^	COI	
*M. fauchaldi* Glasby & Hutchings, 2010	KX172165 ^2^	COI	
*M. kristiani* Zanol, da Silva & Hutchings, 2017	KX172141 ^2^	COI	
*M. mossambica* (Peters, 1854)	KX172164 ^2^	COI	
*M. mullawa* Hutchings & Karageorgopoulos, 2003	KX172166 ^2^	COI	
*M. pseudosessiloa* Zanol, da Silva & Hutchings, 2017	KY605405 ^3^	COI	
*M. victori* sp. n.	MG384997	COI	MNHN-IA-TYPE 1803
*M. victori* sp. n.	MG385000	16S	MNHN-IA-TYPE 1803
*M. victori* sp. n.	MG384998	COI	MNHN-IA-TYPE 1804
*M. victori* sp. n.	MG385001	16S	MNHN-IA-TYPE 1804
*M. victori* sp. n.	MG384999	COI	MNHN-IA-TYPE 1806
*M. victori* sp. n.	MG384996	COI	W.49048

^1^ Sequences from Zanol et al. (2010)

^2^ Sequences from Zanol et al. (2016)

^3^ Sequences from Zanol et al. (2017)

Pair-wise Kimura 2-parameter (K2P) genetic distance and maximum likelihood tree using K2P model and non-parametric bootstrap branch support (1000 replicates) were performed using MEGA version 7.0.26. Tree-based analysis was obtained with all *Marphysa* species having COI sequences available in GenBank and considering other genera of Eunicidae as outgroup (Table 1).

## Systematics

### Taxonomic Account

#### Family Eunicidae Berthold, 1827

##### Genus *Marphysa* Quatrefages, 1866

**Type species.** *Nereis sanguinea* Montagu, 1813

###### Marphysa victori sp. n.

Taxon classificationAnimaliaEunicidaEunicidae

http://zoobank.org/7643A33E-94ED-4DB2-9D47-20A28E808E30

Figs 2 , 3 , 4

####### Material examined.

Holotype: AM W.49047, complete, with 470 chaetigers, ~300 mm long, with a length through chaetiger 10 of 12 mm and width of 13 mm at chaetiger 10 (11 mm without parapodia), regenerating posterior segments. Paratypes: MNHN-IA-TYPE 1805, complete, with 537 chaetigers, 386.06 mm long (approx. 77 cm live), with a length through chaetiger 10 of 15.32 mm and a width of 9.17 mm at chaetiger 10 (7.74 mm without parapodia); MNHN-IA-TYPE 1803, complete, with 414 chaetigers, 255.25 mm long, with length through chaetiger 10 of 13.70 mm and width of 10.76 mm (8.46 mm without parapodia) at chaetiger 10; MNHN-IA-TYPE 1804, complete, with 260 chaetigers, 113 mm long, with length through chaetiger 10 of 9.28 mm and width of 6.68 mm (5.9 mm without parapodia) at chaetiger 10; MNHN-IA-TYPE 1806, complete, with 307 chaetigers, 211.3 mm long, with length through chaetiger 10 of 13.17 mm and width of 8.31 mm (6.93 mm without parapodia) at chaetiger 10; MNHN-IA-TYPE 1807, complete (two fragments), with 260 chaetigers, 110 mm long, with length through chaetiger 10 of 9.87 mm and width of 7.85 mm (6.36 mm without parapodia) at chaetiger 10; MNHN-IA-TYPE 1808, complete, with 267 chaetigers, 135 mm long, with length through chaetiger 10 of 8.2 mm and width of 7.40 mm (6.39 mm without parapodia) at chaetiger 10; MNHN-IA-TYPE 1809, incomplete, with 217 chaetigers, 125.3 mm long, with length through chaetiger 10 of 11.53 mm and width of 8.7 mm (7.19 mm without parapodia) at chaetiger 10; AM W.49048, complete with 530 chaetigers, with length through chaetiger 10 of 11 mm and width of 12 mm at chaetiger 10 (10 mm without parapodia), regenerating posterior segments; AM W.49049, complete with ~350 segments, damaged in 3 places, with length through chaetiger 10 of 10 mm and width of 10 mm (8 mm without parapodia. All type material collected from Carret channel, Arcachon Bay, Bay of Biscay, France (44°40'35"N, 1°6'58"W), intertidal in muddy sediments, 20 September 2016, coll. by G. Binois.

####### Description

(based on holotype and paratypes). Live specimens iridescent, dark red with lighter spots, prostomium appendages and parapodia green olive, end of prostomial appendages whitish, branchial filaments red and iridescent. Recently fixed specimens olive-green to brown with lighter spots, prostomium appendages and parapodia pinkish. Preserved holotype with brown mottled pigmentation anteriorly increases in intensity towards prostomium, antennae, and palps whitish.

**Figure 2.**
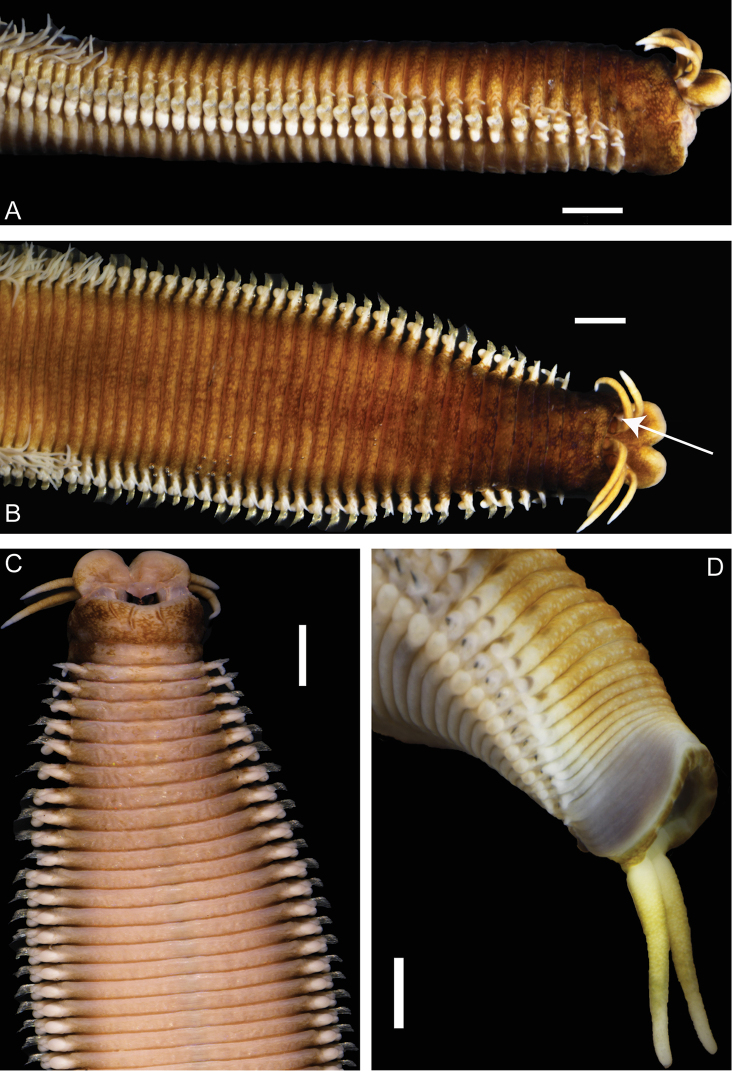
*Marphysa victori* sp. n.: **A** Anterior part, lateral view (paratype MNHN-IA-TYPE 1807) **B** Anterior part, dorsal view (paratype MNHN-IA-TYPE 1807) **C** Anterior part, ventral view (paratype MNHN-IA-TYPE 1807) **D** Pygidium, lateral view (paratype MNHN-IA-TYPE 1803). White arrow showing eye. Scale bars: 2 mm (**A, B, C**), 1 mm (**D**).

**Figure 3.**
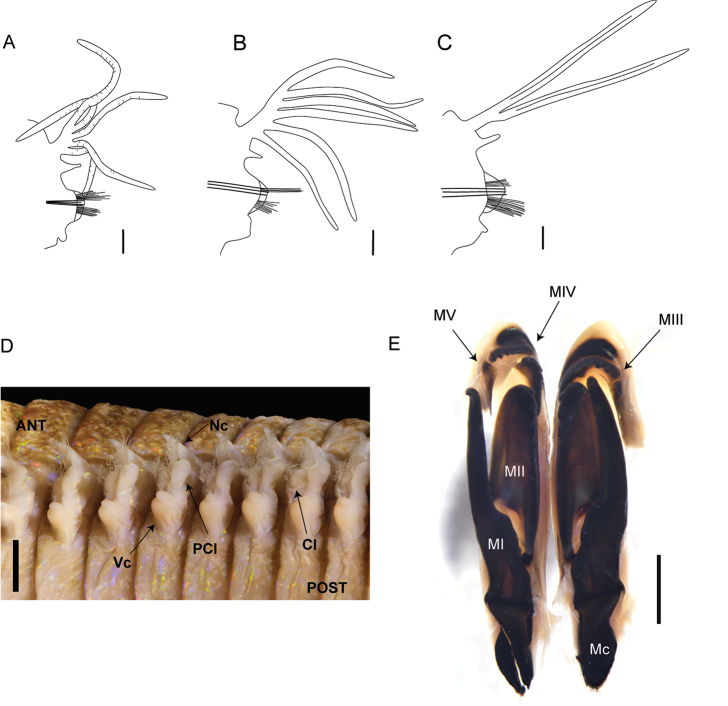
*Marphysa victori* sp. n.: **A** Parapodia from anterior chaetiger (chaetiger 57, paratype MNHN-Arc4) **B** Parapodia from mid-body (chaetiger 127, paratype MNHN-IA-TYPE 1803) **C** Parapodia from posterior chaetiger (chaetiger 352, paratype MNHN-IA-TYPE 1803) **D** Anterior parapodia (from chaetiger 8 to 15), lateral view **E** Maxillae dorsal view (paratype MNHN-IA-TYPE 1809). ANT: anterior part; Cl, Chaetal lobe; Mc, maxillary carriers; MI to MV, maxillae I to V; Nc, notopodial cirri; PCl, postchaetal lobe; POST, posterior part; Vc, ventral cirri. Scale bars: 0.5 mm (**A, B, C**), 1 mm (**D, E**).

Body long, with same width throughout, slightly tapering at anterior and posterior ends. Prostomium shorter than anterior ring of peristomium, as wide as peristomium, bilobed with buccal lips separated by deep ventral and dorsal notch with each lobe rounded with base of them strongly pigmented (Fig. 2A–C). Anterior ring of peristomium longer than posterior ring (2.2 to 3 times) (Fig. 2A, C). Eyes present, positioned between palps and lateral antennae (Fig. 2B), faded in larger specimens (not visible on holotype). Prostomial appendages smooth, arranged in horseshoe, slightly tapering; median antenna longer than lateral antennae, palps shortest appendages (paratypes exhibit considerable variation in the ratio of length of median and lateral antennae and of palps to a lesser extent). Antennal styles and palpostyles smooth although surface slightly wrinkled. MxI more than twice as long as carrier and five times longer than closing system. MxIII at least in part located ventral to MxII. Attachment lamella of MxIII short with irregular shape, placed at the middle of the plate. Left MxIV with attachment lamella semicircular, situated along posterior edge. Right MIV with attachment lamella semicircular, more developed in the central portion, situated along posterior edge. Maxillary formula: I=1+1, II=5+5, III=5–6+6–7, IV=3–4+0, V=1+1 (Fig. 3E).

**Figure 4.**
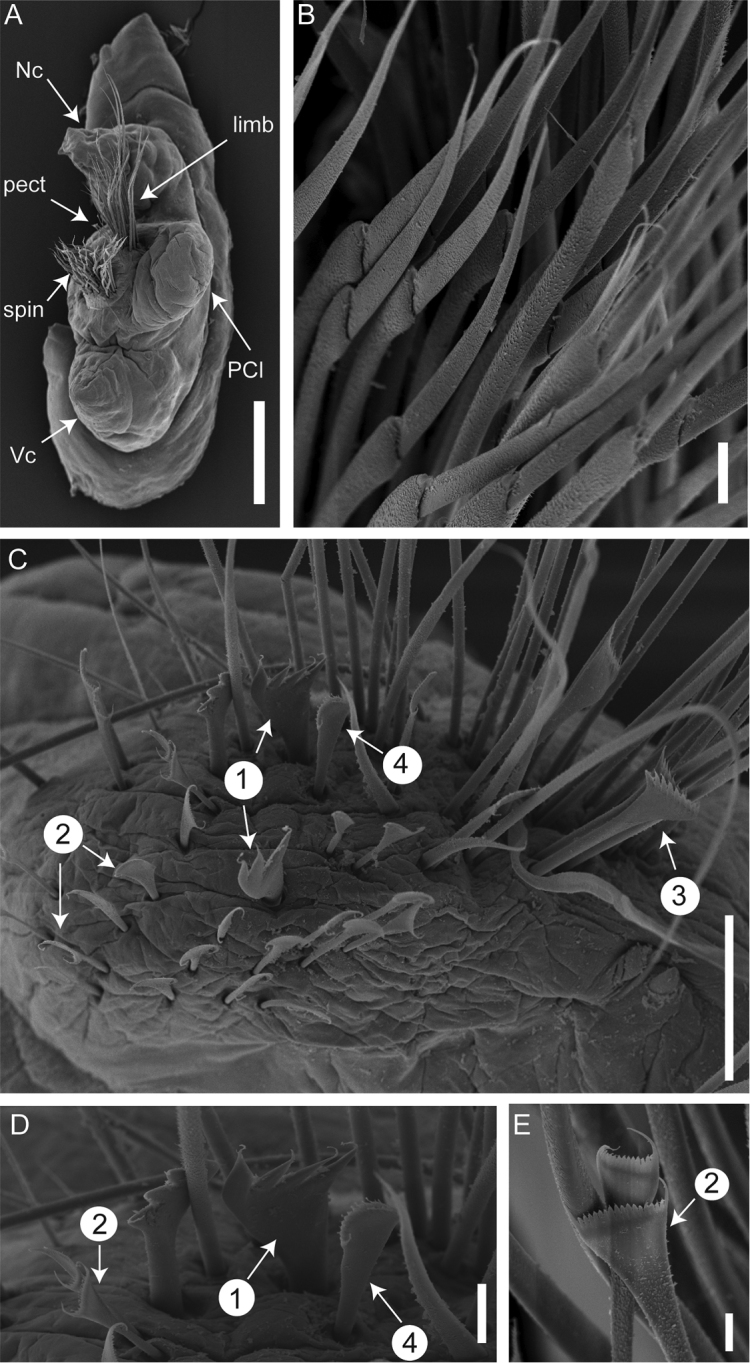
SEM images of *Marphysa victori* sp. n. (holotype AM W.49047): **A** entire lateral view of chaetiger 3 **B** Compound spinigers chaetae (chaetiger 83) **C–D** Different types of pectinate chaetae (chaetiger 243) **E** Isodont, symmetrical chaetae with many teeth (chaetiger 03). Numbers in white circles indicate the type of pectinate chaetae; limb, limbate chaetae; Nc, notopodial cirri; PCl, postchaetal lobe; pect, pectinate chaetae; spin, spiniger chaetae; Vc, ventral cirri. Scale bars: 500 µm (**A**), 200 µm (**B**), 100 µm (**C**), 20 µm (**D**), 10 µm (**E**).

Pre-chaetal neuropodia lobe inconspicuous. Post-chaetal neuropodial lobe conical in the 2–3 first chaetigers, elongate rectangular from chaetiger 4, gradually thereafter becomes wider and rounded; longer than chaetal lobe in anterior chaetigers, shorter in median and posterior chaetigers (Figs 3A–D, 4A). Notopodial cirri triangular, occasionally digitiform in last chaetigers; longer than chaetal lobe in anterior chaetigers, shorter than chaetal lobe in median chaetigers and as long as chaetal lobe in posterior chaetigers (Figs 3A–D, 4A). Ventral cirri from chaetiger 1 to 4–5 conical to tapering, with round wide tips, almost as long as notopodial cirri; basally inflated from chaetiger 5–6, inflated base of round shape with round tip (Figs 3D, 4A), around 1/2 as long as notopodial cirri, gradually decreasing from chaetiger 60 to 110; round with distinct tip from chaetiger 111, around 1/3 as long as notopodial cirri. Ventral cirri as long as or longer than neurochaetal lobe at anterior region (Figs 2A, 3D), slightly shorter to as long as neurochaetal lobe at median and posterior region, sometimes longer than neurochaetal lobe in posteriormost chaetigers.

Branchiae pectinate (Fig. 3A–B), from chaetiger 32, extending posteriorly by last few chaetigers; number of branchial filaments increasing from 3 in first chaetigers to maximum 6 in mid-body, posterior chaetigers with 2 long filaments (Fig. 3C); filaments increasing in size from around 5 times longer than notopodial cirri in anterior chaetigers, around 7 times in mid-body and around 13 times in posterior chaetigers (Fig. 3C), filaments slightly annulated.

Chaetae arranged in two bundles: supra-acicular and sub-acicular, separated by a row of aciculae (Fig. 3A–C). Aciculae dark, with lighter blunt tips, very protruding, 4–6 per parapodium in anterior chaetigers and 2–3 in mid and posterior chaetigers. Subacicular hooks absent. Supra-acicular bundle with limbate and pectinate chaetae; sub-acicular with compound spiniger chaetae (Fig. 4A). Between 25 to 35 limbate chaetae (numbers reducing posteriorly), chaetae of different lengths with hirsute blades, similar to each other. Compound spinigers present, throughout, with more than 40 spinigers within a parapodia, along whole body except last few chaetigers (Fig. 4A, B). Compound falcigers absent. Pectinate chaetae present from chaetiger 2. Up to 34 pectinate chaetae within a parapodia restricted to supra-acicular fascicle of chaetae. Pectinate chaetae of four types. In anterior parapodia, isodont, symmetrical pectinate chaetae (n<10) with 12–16 tapering minute teeth and two long outer winged teeth (nearly 3–4 times longer than inner teeth) (type 2) (Fig. 4C–E). Median parapodia with four types of pectinate chaetae (Fig. 4C–E): isodont, symmetrical pectinate chaetae of type 2; isodont, symmetrical pectinate chaetae with approximately 14 teeth (type 4); anodont, asymmetrical pectinate chaetae with approximately 14 teeth (type 3); anodont, asymmetrical pectinate chaetae with 2–4 large teeth (type 1) (Fig. 4C–E). Posterior parapodia with 3 types of pectinate chaetae: isodont, symmetrical pectinate chaetae with 28 teeth (type 2); anodont, asymmetrical pectinate chaetae with nine parallel teeth (type 3); anodont, asymmetrical pectinate chaetae with 2–4 large teeth (type 1). Subacicular bundle comprising up to 40 compound spiniger chaetae in anterior chaetigers, with surface of blade hirsute (Fig. 4B).

Pygidium with only one pair of long pygidial cirri on ventral margin (approximately as long as last 15 segments), anus slightly crenulated with 12 small indentations (Fig. 2D).

####### Morphological variations.

Paratypes with branchiae starting from chaetigers 26 (MNHN-IA-TYPE 1808) to 28 for smaller specimens and from 28 to 34 for larger ones. Eyes clearly visible on small (MNHN-IA-TYPE 1807 and MNHN-IA-TYPE 1808) and medium specimens, more difficult to see on larger ones. One exceptional specimen (MNHN-IA-TYPE 1805) with one pair of small papillae in addition to one pair of pygidial cirri, with papillae placed more ventrally than cirri.

####### Etymology.

This species is named after Victor Lavesque, first and second authors’ son.

####### Type locality.

NE Atlantic, France, Arcachon Bay.

####### Habitat.

Intertidal on mudflats, under or close to oyster reefs or abandoned oyster farms, 5 to 60 cm depth. Few specimens were found in galleries into old piece of driftwood.

####### Genetic data.

COI gene was successfully sequenced and published at NCBI GenBank for four paratypes: MNHN-IA-TYPE 1803 (accession number: MG384997), AMW.49048 (accession number: MG384996), MNHN-IA-TYPE 1804 (accession number: MG384998) and MNHN-IA-TYPE 1806 (accession number: MG384999). 16S gene was sequenced and published at NCBI GenBank for two paratypes: MNHN-IA-TYPE 1803 (accession number: MG385000) and MNHN-IA-TYPE 1804 (accession number: MG385001) (Table 1).

**Figure 5.**
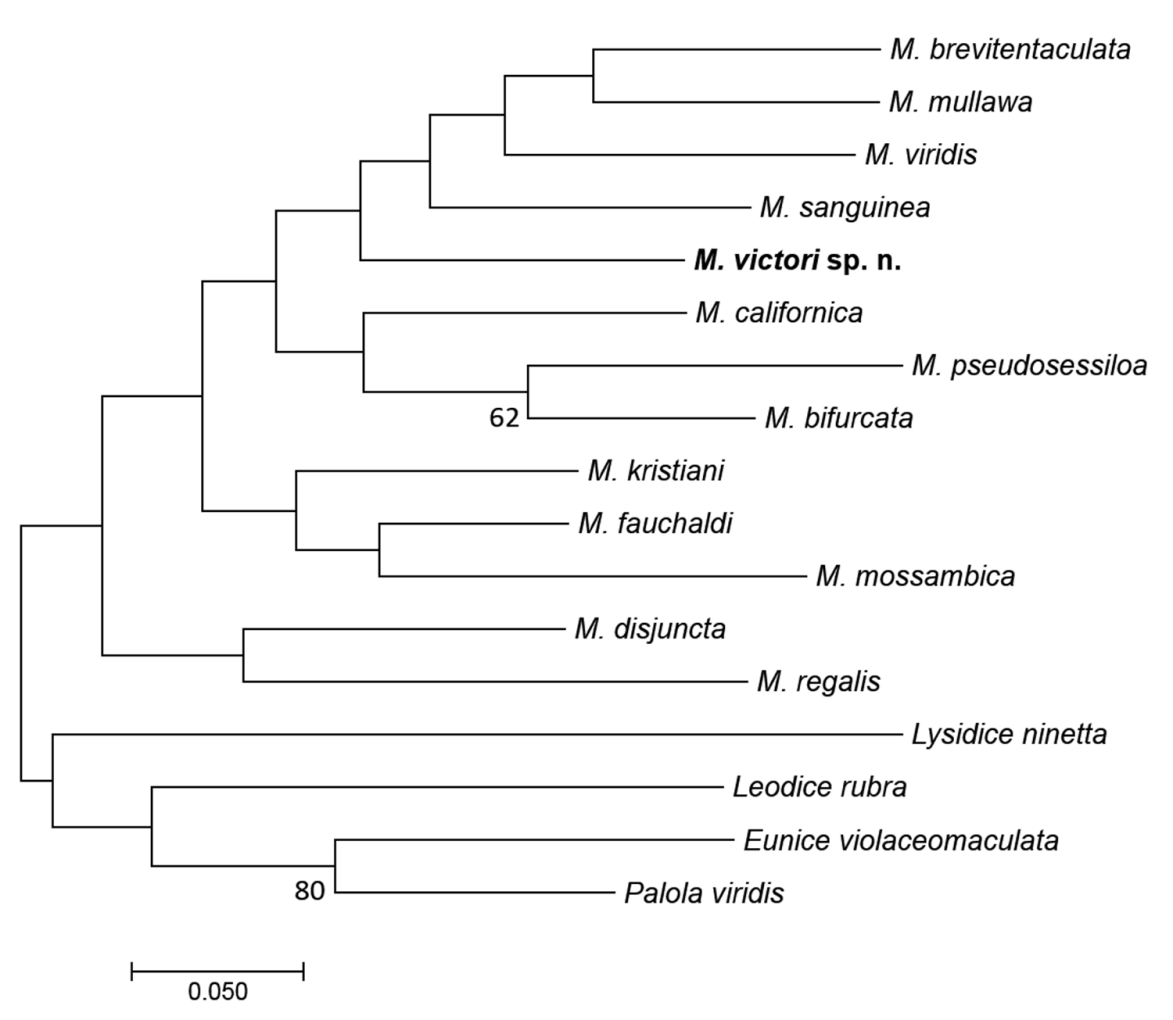
Maximum likelihood tree based on cytochrome oxidase I (COI) sequences and Kimura-2-parameters model. Bootstrap values on nodes if >50.

As the identification of *Marphysa* species from the *sanguinea* group is very complex, molecular tools are very important. First of all, comparison of COI and 16S sequences confirmed that *M. victori* sp. n. was different from *M. sanguinea* (census Zanol et al. 2014) (Fig. 5): interspecific pairwise genetic distances were 21.8% for COI and 15.2% for 16S. Secondly, molecular analysis clearly distinguished *M. victori* sp. n. from other species with sequences available in GenBank (Fig. 5). Finally, they permitted us to link morphological differences to age of specimens. Indeed, intraspecific pairwise genetic distance was zero among specimens and allow to conclude that intensity of eye pigments and the segment on which the branchiae first appear was related to the size (age) of worms and not to the presence of different species.

## Discussion

*Marphysa sanguinea* (Montagu, 1813) is the type species of the genus and has been widely reported from around the world. This is partly because the original description was very brief and poorly illustrated, and because all species superficially look similar. Hartman (1959) complicated the issue by synonymising several species with *M. sanguinea* with no explanation and the species joined a list of so-called “cosmopolitan species” (Hutchings and Kupriyanova 2017). Hutchings and Karageorgopoulos (2003) while trying to resolve the true identity of a commercially important species in Moreton Bay (Queensland, Australia) which had always been called *M. sanguinea*, examined material from SW England and designated a neotype of *M. sanguinea* and then redescribed the species. This then allowed the species from Moreton Bay to be described as a new species, and subsequently other species of *Marphysa* were described from along the east coast of Australia (Zanol et al. 2016, 2017). In the same way, a new species of the *sanguinea* group from French Atlantic coasts is described in this study. In Europe, only two species belong to the B2 group (Fauchald 1970). *Marphysa victori* sp. n. can be distinguished from *M. sanguinea* in having branchiae from chaetiger 26–34 (instead of 13–27), pectinate chaetae of four types (instead of two types, with an absence of anodont, asymmetrical pectinate chaetae with 2–4 large teeth), lacking subacicular hooks (see key above). Moreover, the neotype of *M. sanguinea* was described from south coast of England living intertidally in rocks which are easily identified and which are in a very different habitat to that of *M. victori* sp. n. (mudflats).

Three species are known to occur in Arcachon bay: *M. bellii*, *M. fallax*, and *M. sanguinea*. In the absence of specimens stored in a collection, it is very difficult to know how long *M. victori* sp. n. has been present in Arcachon Bay and confused with *M. sanguinea*. Moreover, the hypothesis that this new species is a non-indigenous species (NIS) cannot be completely dismissed. Arcachon Bay is one of the major French oyster farming sites with a production of 7,000–8,000 t per year of exotic Pacific cupped oyster *Crassostrea gigas* (Thunberg, 1793). In the early 1970s, the Portuguese cupped oyster *Crassostrea angulata* (Lamarck, 1819), which has been farmed in the bay since the end of the 19^th^ century, was decimated by a viral disease (Goulletquer et al. 2002). To sustain the local oyster industry, the exotic Pacific cupped oyster *C. gigas* was then introduced into Arcachon Bay between 1971 and 1975, as spat from Senday Bay, NE Honshu Island, Japan (1,176 t of spat collectors from 1971 to 1975) and as adults from British Columbia, Canada (137.5 t from 1971 to 1973) (Grizel and Héral 1991). Several non-indigenous species, probably introduced with oyster transfers, were recently found in Arcachon Bay (see references in Gouillieux et al. 2016). As *M. victori* sp. n. is very abundant close to oyster reefs, specimens could have been hitchhiked in oyster shells coming from Japan or Pacific coasts of USA. Worldwide, among the B2 group, only two species are characterized by the presence of four types of pectinate chaetae: *Marphysa multipectinata* (Liu, Hutchings & Sun, 2017) recently described from south coast of China and *M. victori* sp. n. *Marphysa multipectinata* differs from *M. victori* sp. n. in the appearance of pectinate chaetae (from chaetiger ~ 70 instead of chaetiger 2 for *M. victori* sp. n.), the maximal number of spiniger compound chaetae (27 vs >40 for *M. victori* sp. n.) and pectinate chaetae (22 vs 34 for *M. victori* sp. n.), the presence of subacicular hooks, the absence of inflated base of ventral cirri, the number of teeth of Mx II (3+3 vs 5+5 for *M. victori* sp. n.) and the presence of two pairs of pygidial cirri (instead of one single pair for *M. victori* sp. n.).

Alternatively, *M. victori* sp. n. could be native from Arcachon Bay and subsequently have been introduced into other European localities. Indeed, local oyster farmers often transfer their spat and juveniles between rearing areas in France (both on the Atlantic and Mediterranean coasts) (Goulletquer et al. 2002) or even to other European countries (Gouillieux et al. 2016). Moreover, thousands (probably millions) of specimens of *M. victori* sp. n. are shipped alive with litter to resellers situated on the western French Mediterranean coasts and then are used as bait by anglers. Recent studies have highlighted the possibility of these practises facilitating the introduction of invasive species (Olive 1994). Bait worm packaging are considered to be an important vector for transporting non-native algae, microorganisms and other invertebrates (Haska et al. 2012; Fowler et al. 2016). For example, a total of 114 taxa have been identified by Fowler et al. (2016) in baitworm shipments from Maine. Moreover, use of live baits contributes to the dispersal of worms in new marine ecosystems. Kilian et al. (2012) showed that a certain number of anglers in Maryland (USA) released their unused baits into the water at the end of a fishing trip. In this way, *M. victori* sp. n. might become a non-indigenous species in the Mediterranean Sea. Finally, the presence of *M. victori* sp. n. in driftwood could also lead to an extension of its geographical distribution in the Bay of Biscay via water currents.

To conclude, we suggest that all records of *Marphysa* from northern Europe need to be carefully checked to see if they represent a currently known species including *M. sanguinea* or represent an undescribed species. As well, the pattern of chaetal arrangement along the body need to be examined under the SEM, and combined with molecular data to correctly identify these species which often superficially resemble each other and vouchers need to be deposited in a museum.

## Key to the European species of *Marphysa*

**Table:** 

1	Compound spinigers only	**2**
–	Both compound falcigers and spinigers	**4**
2	Branchiae limited to anterior chaetigers	***M. kinbergi* McIntosh, 1910**
–	Branchiae present over most of the body	**3**
3	Branchiae from chaetigers 13 to 27, absence of anodont, asymmetrical pectinate chaetae with 2–4 teeth, subacicular hooks present	***M. sanguinea* (Montagu, 1813)**
–	Branchiae from chaetigers 26 to 34, presence of anodont, asymmetrical pectinate chaetae with 2–4 teeth, no subacicular hooks	***M. victori* sp. n.**
4	Branchiae with up to 2 filaments	***M. fallax* Marion & Bobretzky, 1875**
–	Branchiae with 6 or more filaments	**5**
5	Compound spinigers limited to anterior 1/3 or less	***M. bellii* (Audouin & Milne Edwards, 1833)**
–	Compound spinigers along nearly entire body	***M. totospinata* Lu & Fauchald, 1998**


## Supplementary Material

XML Treatment for Marphysa victori
